# Multi-omics analysis of associations between host demographics and saliva metabolome, sugar profiles, and microbiome profiles

**DOI:** 10.1038/s41598-026-44287-w

**Published:** 2026-03-25

**Authors:** Stefania Noerman, Anders Esberg, Carina I. Mack, Hany Ahmed, Björn Egert, Elise Nordin, Carl Brunius, Kati Hanhineva, Ingegerd Johansson, Rikard Landberg

**Affiliations:** 1 Division of Food and Nutrition Science, Department of Life Sciences, Chalmers University of Technology, Gothenburg, Sweden; 2 Department of Odontology, University of Umeå, Umeå, Sweden; 3 Department of Safety and Quality of Fruit and Vegetables, Max Rubner-Institute, Karlsruhe, Germany; 4 Department of Life Technologies, Food Sciences Unit, University of Turku, Turku, Finland; 5 Present Address: Clinical Nutrition Research Centre (CNRC), Singapore Institute of Food and Biotechnology Innovation (SIFBI), Agency for Science, Technology and Research, 14 Medical Drive, MD6, #07‑02, 117599 Singapore, Singapore

**Keywords:** Saliva, Metabolome, Microbiome, Sugar profile, Host demographics, Biomarkers, Microbiology

## Abstract

Omics profiling of saliva is an emerging research area with potential to uncover molecular signatures associated with oral and systemic health. We conducted a comprehensive multi-omics analysis of saliva to investigate associations between host demographics (age, sex, body mass index (BMI)) and molecular profiles. Saliva from 423 participants (16–79-years-old) were analyzed using LC-MS metabolomics (9,380 metabolite features for 416 participants), GC×GC-MS sugar profiling (69 sugars for 200 participants), and full-length 16S rDNA sequencing (500 microbial species for 420 participants). We used random forest modeling, multivariate OPLS analysis, and partial correlation networks for data integration. Age emerged as the strongest demographic factor, explaining up to 30% of variance in metabolite features, 17% in sugars, and 25% in microbial species, while sex showed moderate and BMI minimal associations. Age-associated metabolites included caffeine and trigonelline (higher in older participants) and urocanic acid (higher in younger participants). Younger participants had greater abundance of saccharolytic, facultative anaerobic bacteria while older participants had more anaerobic species. Species in the *Streptococcus*,* Prevotella*, and *Veillonella* genera correlated strongly with salivary sugars. These findings demonstrate that saliva provides a rich source of molecular information related to the individual, and that demographic factors must be considered in saliva-based biomarker-discovery studies.

**Supplementary Information:**

The online version contains supplementary material available at 10.1038/s41598-026-44287-w.

## Background

Biofluid-based metabolomics has emerged as a powerful approach for identifying biomarkers of both exposures and disease outcomes^1,2^. Similarly, microbiome studies have unveiled the crucial role of bacteria in host metabolic regulation^3^. While blood, urine, and feces are commonly studied biospecimens, there is increasing interest in saliva as a non-invasive source of biomarkers for diseases, lifestyle habits, and drug use due to its accessibility and relative ease of collection^4^. Although research into the saliva metabolome and oral microbiome is still nascent, certain features have been associated with drug use and both proximal and distant diseases^5,6^. However, proposed candidate biomarkers often have low specificity, posing challenges for their clinical application. The integration of multiple omics approaches, such as metabolomics, microbiomics, may provide a more comprehensive and sensitive phenotypic profile, enhancing the precision in treatment decisions and interventions.

Whole saliva is a complex biofluid, including salivary gland secretions, blood filtrate, epithelial cell and microbial products, as well as exogenous compounds from food and oral care products. On average, saliva flow varies from 0.1 mL/min (resting state) to 1.5 mL/min (stimulated state). It contains thousands of glycosylated and non-glycosylated proteins and lipids^7^ along with a rich array of small molecules of systemic or local origin^8^. Further, the oral cavity harbors the second largest microbiome in humans, with over 700 bacterial species inventoried so far^9^ and which contribute to the salivary metabolite profile through their metabolic activities. Despite this complexity and potential for biomarker discovery, the saliva metabolome remains substantially less characterized than the saliva proteome or the metabolomes of other biofluids like blood or urine.

Metabolites produced by oral microorganisms not only influence the local environment but may also affect systemic physiology by triggering inflammation and other cell signaling mechanisms^10,11^. Key microbially-produced compounds include trimethylamine N-Oxide (TMAO), short-chain fatty acids (SCFAs), nitric oxide, hydrogen sulfide, cytokines, lipids, and various sugar metabolites^12,13^. As a gateway to the gastrointestinal tract, the oral microbiome has been implicated in an “oral-gut axis” with potential implications for systemic health^14^. However, the few existing multi-omics studies of saliva show inconsistent results regarding both baseline profiles and factors influencing their relationships^15–18^.

Despite the recognized potential of saliva as a biofluid for health research, the impact of fundamental host demographics on the integrated saliva metabolome and microbiome has been inadequately characterized. Previous studies have examined demographic associations with either saliva metabolome or microbiome separately, and often in specific disease contexts or limited age ranges, but comprehensive multi-omics analyses in healthy populations spanning a wide age range are lacking. To address this gap, we conducted a multi-omics study with the overall aim to establish baseline information to inform future saliva-based biomarker research by (1) characterizing associations between host demographics (age, sex, body mass index (BMI)) and saliva LC-MS metabolome, sugar profiles, and microbiome; (2) determining the relative importance of these demographics across the different molecular layers; and (3) identifying potential interactions between the microbial community and metabolite features in host demographics prediction.

## Methods

Details of analytical procedures for sugar, metabolome, and microbiome profiling are described in the Online Supplemental Methods.

### Study population and population characteristics

**Fig. 1:**
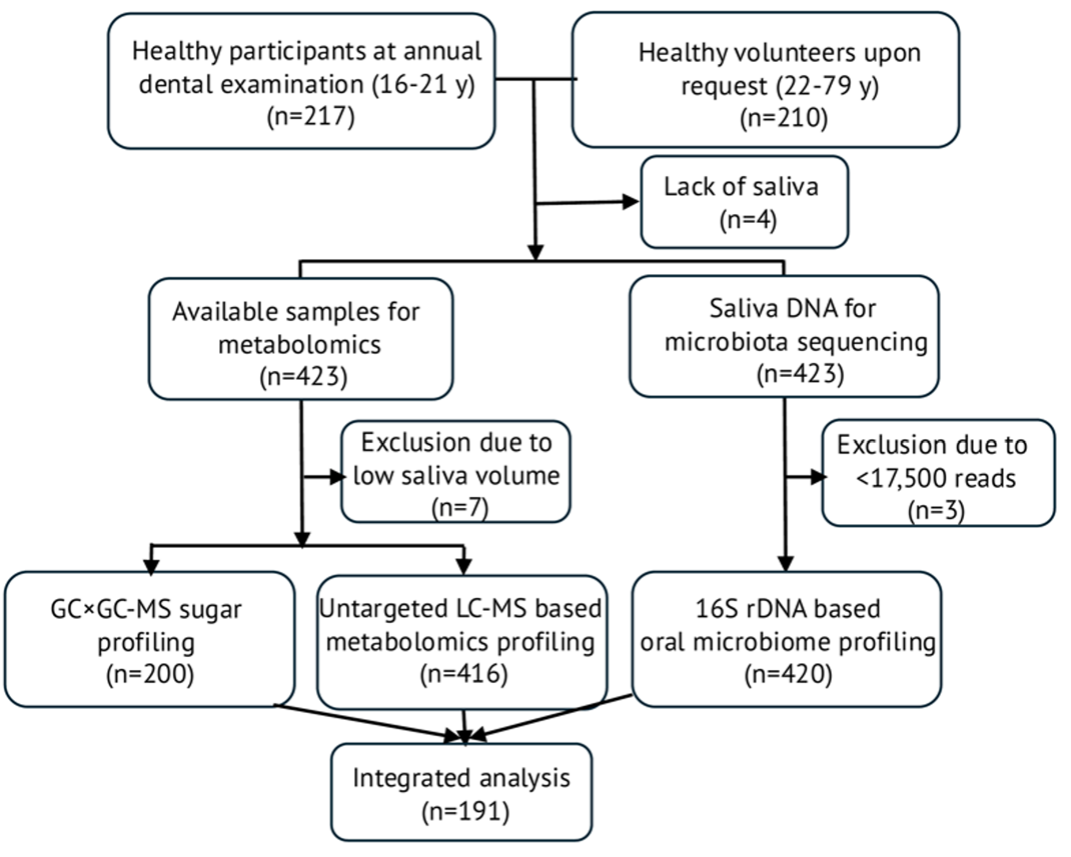
Flowchart showing participant recruitment, exclusion criteria, and sample allocation across the three omics platforms. Numbers in boxes indicate participants included in each stage. LC-MS, liquid chromatography-mass spectrometry; GC×GC-MS, comprehensive two-dimensional gas chromatography-mass spectrometry.

Adolescents and young adults aged 16–21 years scheduled for their free, annual dental check-up at a clinic in Umeå, Sweden, were consecutively invited to complete a questionnaire and provide a saliva sample during their visit (*n* = 217). Additionally, a convenience sample of adults aged 21–79 years residing in northern Sweden volunteered to complete the same questionnaire and donate saliva (*n* = 210). The inclusion criteria required participants to report being healthy and have no antibiotic use within the past month at the time of enrollment. Of the 427 participants included, four were excluded due to insufficient saliva, leaving 423 participants in the study (Fig. 1).

### Saliva collection

Whole stimulated saliva was sampled by two trained dentists according to a standardized routine. The participants had been instructed not to brush or floss their teeth the morning of the saliva collection and not to eat or drink anything apart from still water for at least 3 h before sampling. Samplings were performed between 10 a.m. and 2 p.m. Before collection started, the participants chewed a 1 g piece of paraffin wax to softness, spat out the produced saliva, and continued to chew for 3 min while frequently spitting saliva into a funnel placed in an ice-chilled sterile test tube. Samples were stored on ice until transferred to – 80 °C within 2 h.

### Information on lifestyle factors

The participants completed a questionnaire that included questions on highest attained education level, height, body weight, health status (including gum bleeding), current medication (including antibiotic use), and lifestyle habits (physical activity, diet, oral hygiene, tobacco use (smoking and use of Swedish snus (oral moist snuff)) (Supplementary Table 1). Diet was recorded using a semi-quantitative food frequency questionnaire (FFQ) designed to reflect habitual intake over the past 12 months. The FFQ comprised 93 food items/aggregates and was a slightly reduced version of the validated FFQ2020 instrument^19^. Tobacco use was recorded as never, ex-, or present use. Habitual oral hygiene was assessed by asking for daily toothbrushing frequency, and whether dental floss or fluoride-containing products (toothpaste, mouth rinse, and other products) were used or not, together with the participants’ self-reported caries susceptibility. For antibiotic use, 120 participants answered, “I do not know”, which was coded as no use, and all participants were left in the analyses as none had an ongoing or recently completed treatment (see inclusion criteria) combined with research reporting oral microbiota to be restored within 4 weeks after antibiotic exposure^20^.

### LC-MS metabolite profiling

LC-MS metabolomics analysis was performed for 416 saliva samples, 7 participants had insufficient sample volume and were thus omitted (Fig. 1). The analyses were run at the Food Sciences Unit, Department of Life Technologies, University of Turku (Turku, Finland). In brief, LC-MS grade acetonitrile was used for protein precipitation and metabolite extraction. The samples were analyzed by a UHPLC-qTOF-MS system (Agilent Technologies, Waldbronn, Germany) using both reversed phase (RP) and hydrophilic interaction (HILIC) chromatographic separation techniques in both positive and negative ionization modes. Data acquisition was performed using the vendor’s software. Peak picking and alignment of molecular features were performed using MS-DIAL v.4.92. Preprocessing of metabolomics data was performed using the notame v. 0.2.0^21^ R package in R v.4.0.3^22^. Details regarding the data acquisition and preprocessing steps are available in the Online Supplemental Methods. To remove potentially irrelevant features, metabolome data were filtered by removing features with *m/z* > 1000 and retention time < 0.8 min, which reduced the numbers of features from 12,673 to 9,380. Imputation of missing values for LC-MS metabolomics data was performed during preprocessing using the notame package. Data were log-transformed, centered and auto-scaled to standard deviation to account for heteroscedasticity. Metabolites were identified by manual inspection of the molecular characteristics (*m/z*, retention time and the MS/MS spectrum) against an in-house spectral library and online databases such as MoNA (https://mona.fiehnlab.ucdavis.edu/), HMDB and LipidMaps^23^. Features with matching *m/z*, retention time, and MS/MS spectra with the standard data in the in-house spectral library were labeled as level I while matches against online databases were labeled as level II according to the reporting guidelines from Metabolomics Standard Initiative^24^. Annotation to level III to certain compound groups based on typical fragmentation patterns was performed with experts’ knowledge, aided by SIRIUS^25^ and MSFinder^26,27^. Unknown features were labeled as level IV. Data were log-transformed, centered and scaled to standard deviation.

### Sugar profiling

GC×GC-MS (Shimadzu GCMS QP2010, Kyoto, Japan) profiling was performed for 200 randomly selected samples out of the 423 samples for workload reasons. The analyses were performed at the Max Rubner-Institute, Karlsruhe, Germany. Briefly, proteins were precipitated with cold methanol, followed by evaporation and a two-step derivatization procedure (methoximation and trimethylsilylation). Details regarding measurement parameters and sample preparation can be found in the Online Supplemental Methods. Internal standards with 23 isotopically labeled sugar compounds in concentrations expected in saliva were used in conjunction with 7 calibration levels yielding calibration curves ranging from 0.01 to 250 µmol/L for absolute quantification of 39 sugar compounds (Supplementary Table 2, Supplemental Methods). Automated integration was performed using AnalyzerPro XD v1.16.8 (SpectralWorks Ltd., Runcorn, UK; https://spectralworks.com/software-2/analyzerpro-xd/) followed by alignment and demodulation using our in-house R-modules in R v4.3.3 (see Online Supplemental Methods). After correction for drift and matrix effects using internal standards, values were either used as-is for relative quantification, or as absolute concentrations estimated from the best-fitting regression within the linear range of the calibration curve.

Due to problems with the equilibrium between sugar acids and corresponding lactones, the sugars erythronic acid, arabonic acid, and ribonic acid were excluded. Additionally, the quantities of fructose, lactose, and maltitol fell outside the calibration range for many samples and were estimated as relative amounts. Hence, 33 sugar compounds with absolute quantitative values were kept for data analysis. Among 40 relatively quantified sugar compounds, mannoheptulose was excluded due to technical issues, and 6 sugars (pinitol, galactitol, galacturonic acid, melibiose, an unknown inositol and an unknown disaccharide) were excluded due to > 35% missing values and uncertainty in missing value imputation. Including fructose, lactose and maltitol, a total of 36 sugar compounds with relative quantities were accepted for data analysis (Supplementary Table 2).

Among the 69 sugar compounds remaining for downstream analyses (Supplementary Table 2), 48 were identified from reference substances or an in-house database (MSI level I), 2 were identified from spectral libraries (Fiehn, NIST17; MSI level II), and 19 unknown sugar compounds were identified based on characteristic mass spectra with typical mass fragments for sugar compounds, which could be assigned to a sub-class of sugar compounds such as sugar acids or polyols (MSI level III). Imputation of missing values was performed using a random forest algorithm (mvImpWrap function in StatTools package v0.0.916)^28^, and data were log-transformed, centered and scaled to standard deviation.

### Oral microbiota characterization

The oral microbiota was characterized from saliva extracted DNA and full 16S rRNA gene sequencing. Briefly, DNA was extracted using the GenElute™ Bacterial Genomic DNA kit (Sigma-Aldrich, St. Louis, MO, USA) with lysozyme, mutanolysin, and Proteinase K and was treated with RNase and purified as described previously^29^. The entire 16S rRNA gene was PCR amplified, and after confirmation of a single fragment of the expected 1,465 bp, amplicons, purified and quantified. Library preparation included barcoding of the amplicons using the Native Barcoding Kit 96 V14 (SQK-NBD114.96, Oxford Nanopore Technologies, Oxford, UK; https://store.nanoporetech.com/native-barcoding-kit-96-v14.html), followed by pooling and cleaning using 0.4x AMPure XP Beads, and finally fusing of the Native Adapter using T4 DNA Ligase (NEB, E6056). After additional cleaning and quantification, samples were loaded on a pre-primed R10.4.1 flow cell (Oxford Nanopore Technologies) and sequenced using a GridION nanopore sequencer (Oxford Nanopore Technologies) for 72 h. Base-calling of nanopore signals and demultiplexing were performed on the GridION using the MinKNOW (Oxford Nanopore Technologies), Dorado base callers Super accurate (SUP) model and Porechop (v0.2.4, https://github.com/rrwick/porechop) generating demultiplexed FastQ files, with a quality score ≥10 with a read length between 1,350 and 1,800 bp. A commercial mock community (ZymoBIOMICS Microbial Community DNA Standard, D6305, NordicBiosite, Stockholm, Sweden) with pre-purified DNA was used as a positive control, and ultrapure water as negative control.

The EMU algorithm was employed for taxonomic annotation against the eHOMD 16S rRNA database^30,31^. Three samples were excluded due to read counts < 17,500, leaving 420 participants with microbiome data. For calculation of the Shannon α-diversity index, read counts were rarefied to a sequencing depth of 17,500 reads per sample to control for differences in library size, as Shannon diversity is sensitive to sequencing depth and evenness. For all other analyses, reads were normalized using total sum scaling (relative abundance), followed by addition of a small pseudo-count (minimum non-zero value divided by 1,000), log-transformation, centering, and scaling to unit variance. This approach avoids data loss from rarefaction while providing appropriate normalization for multivariate and regression-based analyses of compositional microbiome data (Supplementary Table 3).

### Statistical analyses

First, ANOVA was used to assess the proportion of variance in LC-MS features, sugars, and microbial taxa that was explained by demographic characteristics (age, sex, and BMI). The proportion was approximated as the type III sum of squares divided by total sum of squares.

Second, we employed random forest (RF) to identify features-of-interest associated with each demographic factor using the R package MUVR*2* v0.1^32^, which conducts modeling within a nested cross-validation framework to minimize overfitting and false discovery. The MUVR2 procedure also performs recursive feature elimination within the nested cross-validation loops to achieve an unbiased selection of variables-of-interest. RF was performed using the settings nRep = 50, nOuter = 8, varRatio = 0.90 for the 9,380 LC-MS features (*n* = 416 individuals), 69 sugars (*n* = 200 individuals), and 500 microbiome taxa (*n* = 420 individuals), respectively, as predictor variables and individual demographics, (sex, age, BMI and microbiota α-diversity (Shannon index)) as target variables^33^. Modeling performance was assessed using Balanced error rate (BER) for sex and Q^2^ for the continuous variables age, BMI, and Shannon index. To test the modelling performance, resampling tests were performed on relevant models (BER < 0.4 or Q2 > 0.2, selected heuristically to represent a relevant association effect size) against the null hypothesis (H_0_) distribution built using randomly permuted labels of Y (n_Resample_=100) resulting in a permutation p-value (p_Perm_). The selection of variables-of-interest was performed for models with p_Perm_<0.05.

Third, RF selected variables-of-interest were evaluated in linear regression models with age, sex, or BMI as outcomes, with, based on the outcome, contrasting demographic variables included as covariates. For bacterial species, the MaAsLin3 R framework was used^34^. The MaAsLin3 models included age, sex and sample sequencing depth (sample total number of reads) as covariates and the models were run with a two-part regression strategy, i.e., testing first associations with prevalence (zero versus non-zero carriers), followed by associations to abundance among individuals where the species was present. Data were transformed as described above. For prevalence models, a one-unit change in the outcome variable corresponds to a coefficient change in the log-odds of a feature, and for abundance models, a one-unit change in the outcome corresponds to a two-fold change in the relative abundance of the feature. Adjustment for multiple comparisons was performed using the Benjamini-Hochberg method for false discovery, considering FDR ≤ 0.05 as selection criterion for being in the final selection of variables-of-interest.

Finally, to evaluate a potential synergistic effect, selections of variables-of-interest from the three omics layers and confounder adjusted linear regression were combined and used to predict age, sex and BMI by RF using the settings described above and by OPLS using leave-one-out cross-validation. Model strengths were evaluated as described above. Additionally, partial Spearman correlations were calculated between bacterial species (*n* = 35) and sugars (*n* = 20) derived from the baseline RF models and included age and sex as covariates.

To further investigate whether consumption of caffeine-containing drinks mediated the associations between demographics and caffeine metabolites, counterfactual mediation analysis was performed using the *mediation* package v4.5.0^35^. Age was modelled as independent and age-related metabolites as dependent variables without covariates or with sex, BMI, tobacco use and toothbrushing frequency as covariates. Tested mediators included intake frequency of filtered coffee, boiled coffee, tea, energy drinks, soft drinks, and their combination (total caffein exposure) obtained by multiplying the intake frequency with their caffeine content, i.e., 550 mg/serving for filtered coffee, 375 mg/serving for boiled coffee, 30 mg/serving for tea, 70.5 mg/serving for energy drink, and 24 mg/serving for soft drinks^36–38^. Non-parametric bootstrapping (*n* = 500) was used to estimate confidence intervals.

Furthermore, sensitivity analyses assessing associations between salivary omics profiles and other potentially determining factors, such as tobacco use, habitual oral hygiene, and self-reported caries susceptibility were performed as described above. For the OPLS analyses, the SIMCA P+ (v18.0, Sartorius Stedim Data Analytics AB, Umeå|Malmö, Sweden) was used. All other analyses were performed using R 4.3.1 in the BIANCA cluster at the Uppmax facility, provided by the National Academic Infrastructure for Supercomputing in Sweden (NAISS), specifically designed to analyze sensitive data.

## Results

### Study group

In total, 423 individuals were included in the present study after excluding 4 participants due to insufficient saliva (Fig. 1). The included participants represented a younger (≤ 21 years of age, *n* = 216) and an older (*n* = 207) age group, with 98% having complete LC-MS metabolomics profiles and 99% having complete microbiome profiles. Out of the 423 individuals, 200 randomly selected participants were analyzed for sugaromics profiles (referred to as sugars), of which all yielded successful information (Fig. 1).

There were more females than males in both age groups (Table 1), most were non-tobacco users and had normal weight, though ∼25% were overweight or obese (BMI > 25). One third reported being physically inactive, and most reported daily toothbrushing with a fluoridated toothpaste (Table 1). Self-reported dietary habits of participants were characterized by proportionally higher than recommended fat intake and lower carbohydrate intake (Table 1).

**Table 1:** Demographic and lifestyle characteristics of study participants (*n* = 423). Data are presented as mean with standard deviation (sd) for continuous variables and n (%) for categorical variables.

	≤21 years (*n* = 216)	≥22 years (*n* = 207)
Women, (%)	55.1	69.4
Age (years), mean (sd)	17.8 (1.1)	41.8 (14.9)
BMI (kg/m^2^), mean (sd)	22.4 (3.2)	23.9 (3.0)
Overweight/Obese (BMI > 25), %	18.1	31.5
Highest or ongoing education level, %		
Junior high school	70.0	1.1
High school or equal	29.6	32.9
University	0.5	66.2
General behavior, %		
Current smoker	2.9	1.0
Current Swedish snuff user	7.1	12.2
Never/occasionally physical active, %	35.7	30.0
Physical active 1–3 times/week, %	36.7	45.6
Physical active > 3 times/week, %	27.6	24.4
Diet intake, E%, mean (sd)		
Carbohydrates	39.7 (6.2)	39.8 (6.9)
Sucrose	6.0 (2.5)	5.4 (2.1)
Total fat	44.9 (7.1)	41.9 (7.1)
Protein	14.5 (2.9)	15.7 (2.9)
Caffeine containing products, mean (sd) intakes/day		
Filtered coffee	0.54 (0.95)	1.86 (1.28)
Boiled coffee	0.11 (0.37)	0.11 (0.47)
Tea	0.27 (0.63)	0.60 (0.87)
Energy drinks	0.11 (0.07)	0.02 (0.07)
Sodas	0.21 (0.36)	0.06 (0.09)
Oral behavior, %		
Tooth brushing at least once a day, %	81.8	98.5
Gum bleeding on brushing, %	27.7	14.9
Dental flossing, %	25.1	80.5
Using fluoridated toothpaste, %	91.9	99.5
Using other fluoride preparation, %	6.3	25.5
Reporting caries susceptibility,%	9.3	9.2

### Salivary molecular profiles

Peak-picking from the four analytical modes in the LC-MS metabolomics analysis resulted in a data matrix of 19,674 features. After filtering low-quality features, 12,673 features remained for imputation of missing values. Features with mass-to-charge ratio (mz) < 1,000 and retention time (rt) < 0.8 min were removed, resulting in 9380 features for downstream analyses, out of which 85 were annotated, with 80 unique, after statistical evaluation (Supplementary Table 4).

Sugar profiling resulted in 33 sugars with absolute quantities, and 36 sugars with internal standard calibrated relative quantities as described above. These were forwarded to statistical analyses (Supplementary Table 2).

For the oral microbiome, after exclusion of taxa present in only one participant and samples with fewer than 17,500 reads, the average number of reads per sample was 85,417 (min 17,526; max 220,488). These represented 15 phyla, 131 genera, and 500 named species or unnamed phylotypes. The top three most abundant phyla were *Firmicutes* (52.5% mean relative abundance), *Bacteroidetes* (21.7%), and *Actinobacteria* (13.5%). The genera with a mean relative abundance over 2% included *Streptococcus* (32.5%), *Prevotella* (15.8%), *Veillonella* (9.6%), *Rothia* (7.1%), *Neisseria* (5.0%), *Schaalia* (3.6%), *Haemophilus* (3.5%), *Gemella* (3.4%), *Porphyromonas* (3.3%), and *Granulicatella* (2.9%). The five most abundant species were *Streptococcus salivarius* (8.3%), *Prevotella melaninogenica* (7.2%), *Rothia mucilaginosa* (6.3%), *Streptococcus mitis* (4.2%), and *Veillonella dispar* (3.8%). The detected species appeared in three distinct clusters at the genus level, with one dominated by *Streptococci*, one by *Prevotella*, and one by *Neisseria* (Supplementary Fig. 1). A complete list of detection prevalence and relative abundances is shown in Supplementary Table 3, which also indicates the 30 core species, i.e., present in all participants.

**Fig. 2:**
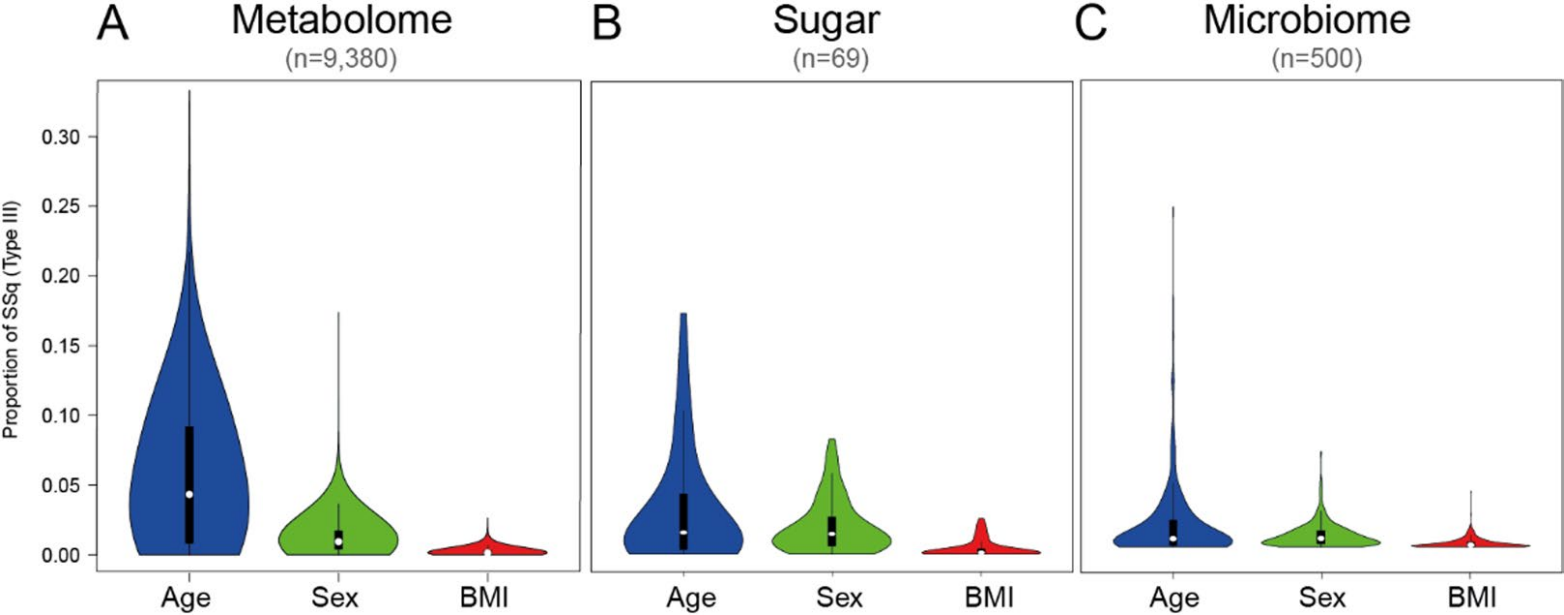
Distribution of variance proportions explained by age, sex, and body mass index (BMI) across molecular features from (**A**) LC-MS metabolomics (*n* = 9,380 features), (**B**) sugar profiling (*n* = 69 compounds), and (**C**) microbiome analysis (*n* = 500 species). Variance proportions were calculated using ANOVA. Violin plots show probability density distributions with median and quartile indicators.

Initial ANOVA analysis showed that age explained the largest proportion of LC-MS feature variance (median around 5% and maximum around 30%), whereas sex and BMI medians were < 1% (Fig. 2A). Similarly, age was the main demographic determinant to explain variance of sugar concentrations (median ∼2.5% and maximum of ∼17%), again followed by sex and BMI (Fig. 2B). Age also accounted for the largest proportion of variance across species abundances of oral microbiota, with a median of ∼1% and a maximum of ∼25%, with sex and BMI being less influential (Fig. 2C).

**Fig. 3:**
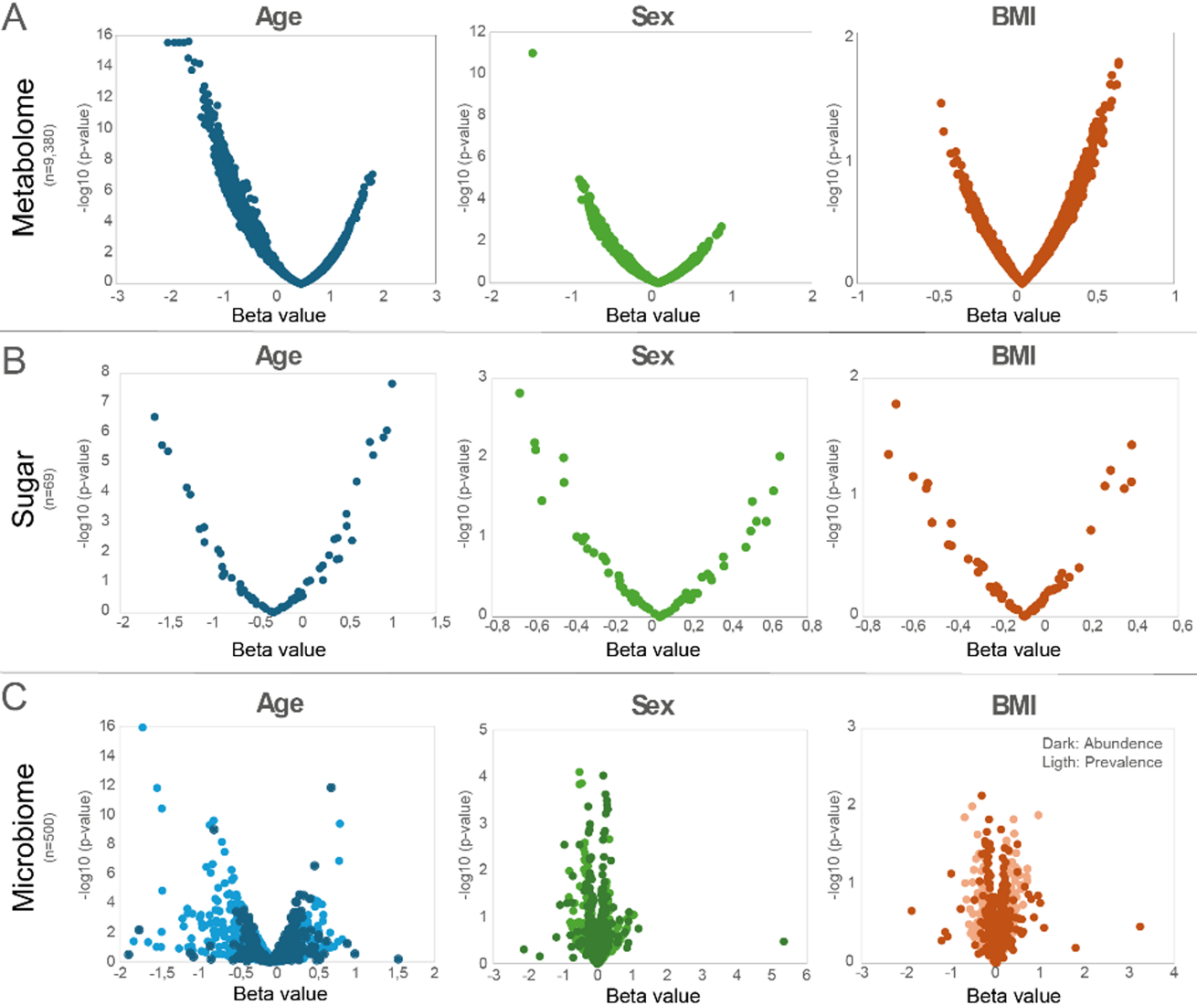
Volcano plots displaying the relationship between effect size (β-coefficients, x-axis) and statistical significance (-log₁₀(p-value), y-axis) for associations between demographic variables and molecular features. (**A**) LC-MS metabolomics features (*n* = 9,380), (**B**) sugar compounds (*n* = 69), and (**C**) microbial species (*n* = 500). Each point represents one molecular feature, with colors indicating different demographic associations. For microbiome data, darker color indicates abundance and lighter color prevalence.

Linear regressions for the 9,380 LC-MS features, 69 sugars, and 500 oral bacterial species, respectively, revealed high variation in β-coefficients and p-values, supporting significant and varying impacts of the evaluated demographics (Fig. 3A–C) calling for detailed analyses of the associations.

### Prediction of age, sex, and BMI by overall LC-MS metabolome, sugars, and microbiome profiles

**Fig. 4:**
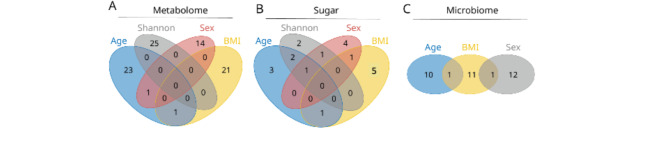
Venn diagrams showing overlap between molecular features selected by random forest modeling as associated with different demographic outcomes. (**A**) LC-MS metabolome (*n* = 85), (**B**) sugars (*n* = 20), and (**C**) microbiome (*n* = 35). Each circle represents features associated with age, sex, BMI, and Shannon diversity index.

Random forest (RF) analyses resulted in 80 unique LC-MS metabolite features (representing 12 identified and 68 unknown compounds) associated with age, sex, or BMI (Supplementary Table 4). Additionally, 20 sugars (Supplementary Table 2), and 35 bacterial species (Supplementary Table 3) associated with one or more of the four tested outcomes, i.e., age, sex, BMI, and Shannon diversity. Notably, the number of shared features across the four outcomes was very low (Fig. 4A–C).

The capacity to predict age, sex, BMI, and bacteria Shannon diversity index from RF-selected LC-MS features, sugar components, and bacterial species of interest, respectively, is reported in Table 2. The LC-MS feature profile provided a stronger association with age (Q^2^ = 0.72) than the sugar or microbiota profiles, whereas sex associated more strongly with microbiome profiles (BER = 0.41) than sugar and metabolite features. BMI could not be predicted by any of the three omics layers (Table 2).

**Table 2:** Random forest modeling performance for predicting age, sex, BMI, and microbial α-diversity (Shannon diversity index) using features selected as variables-of-interest from LC-MS metabolomics (*n* = 85 features from 416 participants), sugar profiling (*n* = 20 compounds from 200 participants), and microbiome analysis (*n* = 35 species from 420 participants).

	*N*	Age		Sex		BMI		Shannon	
	Samples	Q^2^	pPerm	BER	pPerm	Q^2^	pPerm	Q^2^	pPerm
Metabolome	416	0.72	1.1E-18	0.26	9.3E-09	-0.1	NA	0.21	8.7E-19
Sugars	200	0.49	5.8E-19	0.36	3.2E-03	-0.1	NA	0.13	6.8E-09
Microbiome	420	0.53	4.0E-19	0.41	6.3E-02	-0.1	NA	NR	NR

R^2^=proportion of variance explained; Q^2^=proportion of variance predicted in cross-validation; pPerm=statistical significance from RF resampling tests; MR=misclassification rate; BER=Balanced error rate; NA = not analyzed; NR = not relevant due to poor model performance.

### Age and sex associations with LC-MS metabolome features

**Fig. 5:**
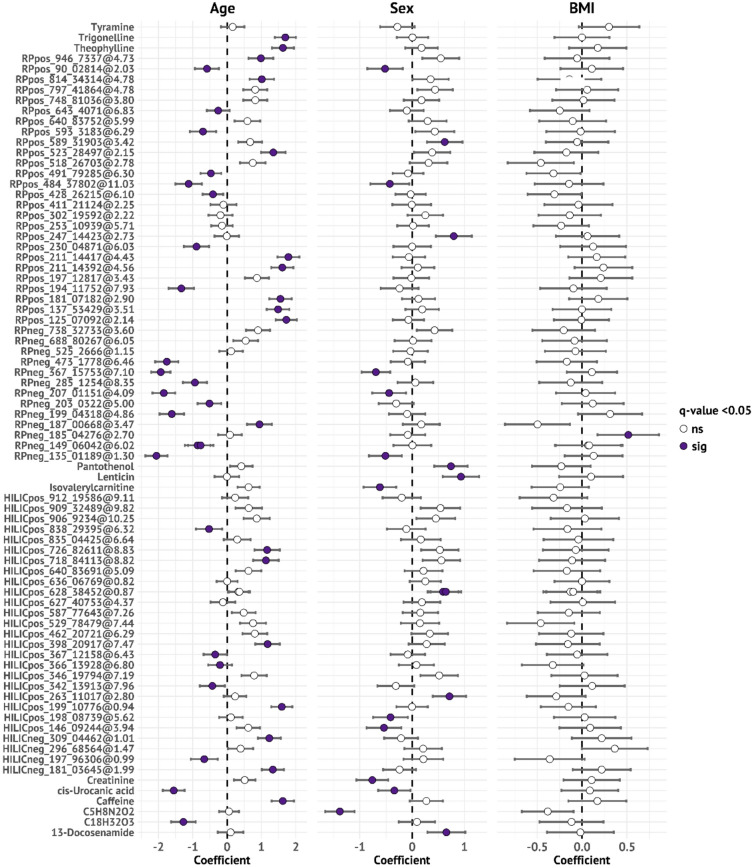
Forest plot showing standardized effect sizes (β-coefficients) and 95% confidence intervals for associations between demographic variables and LC-MS metabolite features selected by random forest analysis (80 features after removing redundant annotations). Results are from covariate-adjusted linear regression models with age, sex (men to the left and women to the right), and BMI as independent variables.

After excluding the 5 LC-MS features that mapped to the same metabolite (Supplementary Table 4), covariate adjusted linear regression identified 42 LC-MS features associated with age after FDR adjustment (Fig. 5). Of these, 24 associated with younger age, including urocanic acid and C18H32O3 (leukotoxin A), and 18 with older age, including caffeine, theophylline, and trigonelline (Fig. 5). Further, 19 LC-MS features associated with sex, with 11 being higher in males, including creatinine, and 8 being higher in females, including the lentil intake marker lenticin (Fig. 5). One undefined feature (RPneg_185.04276@2.70) associated with higher BMI (Fig. 5).

To further explore the identified associations between age and coffee-related metabolites (caffeine, trigonelline, and theophylline metabolites), we examined whether caffeine intake served as a mediating factor in these relationships using mediation analysis. Total caffeine intake, and filtered coffee intake were suggested as potential mediators of the associations (proportion mediated 33–38%). Boiled coffee, tea and soft drinks did not seem to mediate the association (Supplementary Table 5). Inclusion of sex, BMI, tobacco use and frequency of toothbrushing had minimal effects on the estimates with an attenuation on the fourth decimal for all mediators.

### Age and sex associations with sugars

**Fig. 6:**
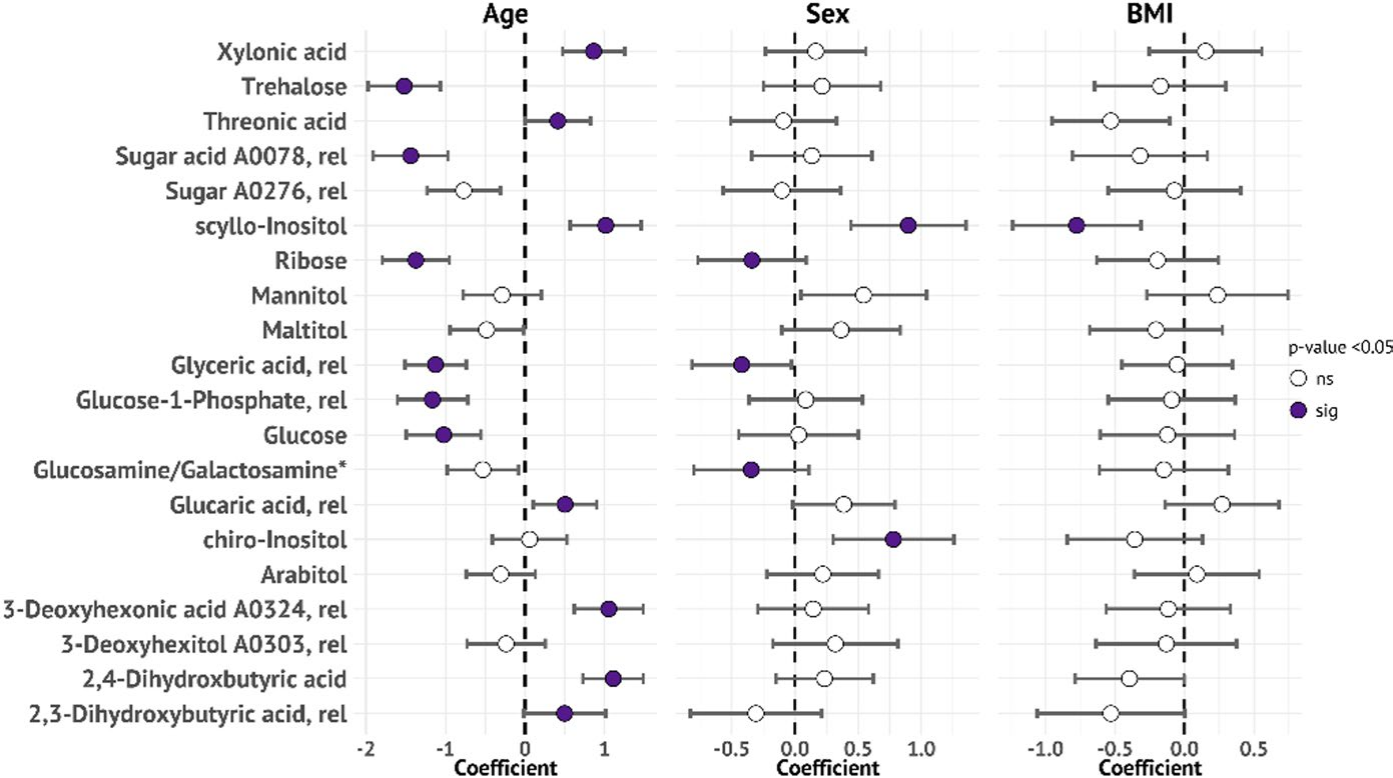
Forest plot showing standardized effect sizes (β-coefficients) and 95% confidence intervals for associations between demographic variables and sugar compounds selected by random forest analysis (*n* = 20 compounds). Results are from covariate-adjusted linear regression models with age, sex (men to the left and women to the right), and BMI as independent variables. Five sugars did not meet the cut-off value for q, i.e., > 0.05, and were not included in the figure. *indicates the sum for glucosamine and galactosamine, which are coeluting.

Age-associated patterns in salivary sugars revealed distinct profiles. Among 20 sugars identified by RF analysis, 13 showed significant age-dependent abundance patterns (*p* < 0.05; Fig. 6). Younger participants (≤ 21 years) had higher levels of trehalose, ribose, glyceric acid, and glucose, all involved in glycolytic processes and with various biological functions. Older participants displayed elevated levels of xylonic acid, a sugar acid implicated in oxidative stress responses^39^; threonic acid; *scyllo*-inositol, a stereoisomer associated with neurodegenerative processes; and dihydroxybutyric acid^40^. Sex differences emerged in 5 sugars independent of age effects. Men showed higher ribose, glyceric acid, and glucosamine/galactosamine levels, potentially reflecting bacterial release from saliva glycoproteins, while women had more *scyllo-* and *chiro-*inositol. These differences may relate to sex-specific hormonal regulation of carbohydrate metabolism, as suggested by population-level studies of glucose homeostasis^41^. BMI demonstrated minimal associations, with only *scyllo*-inositol showing a significant association (Fig. 6). This contrasts with blood metabolome studies, where BMI associations are typically strong for several metabolites, highlighting saliva’s unique metabolic compartmentalization.

### Age and sex associations with saliva microbiota species

**Fig. 7:**
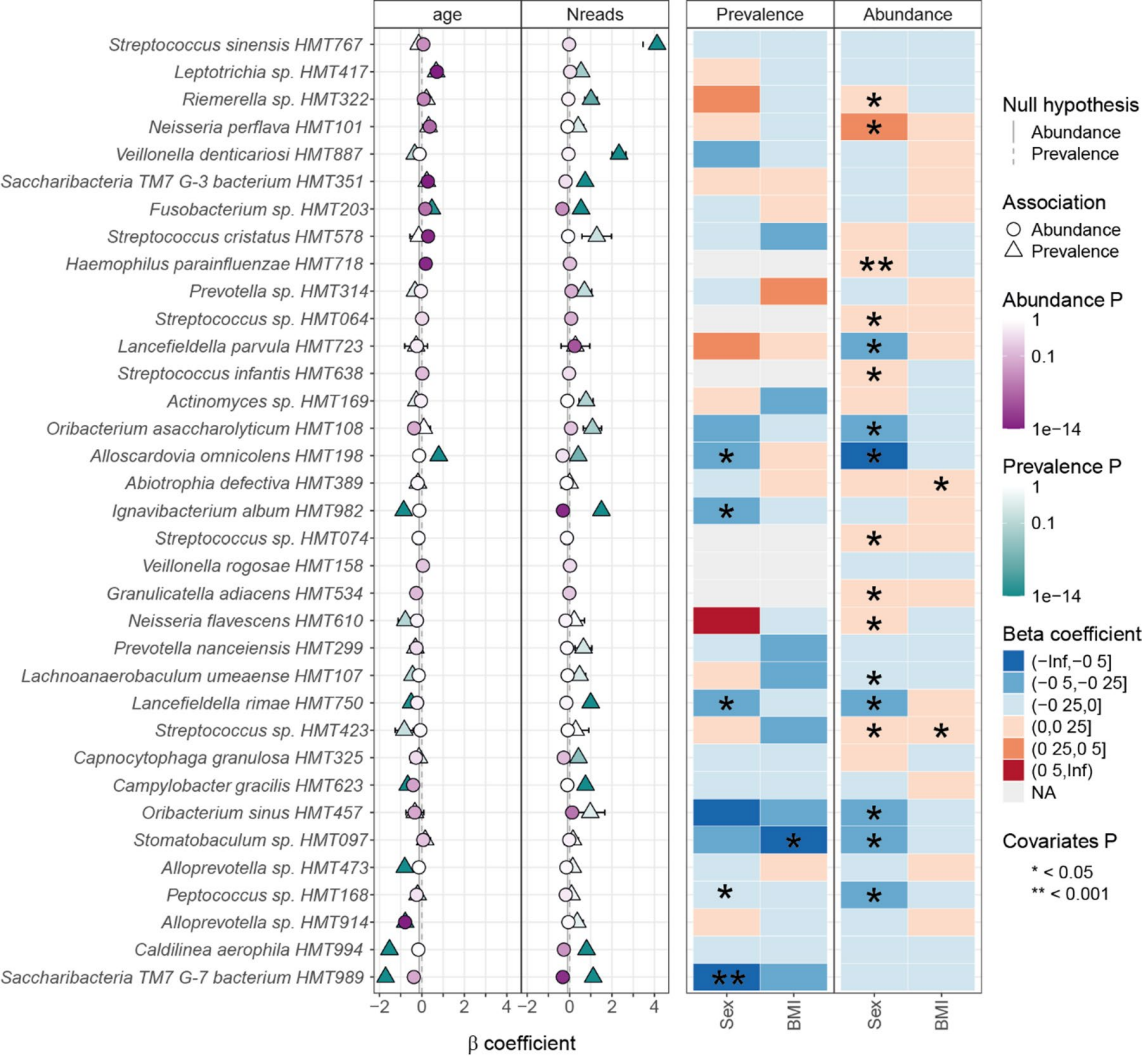
Comprehensive visualization of demographic associations (standardized β-coefficients with 95% confidence intervals) with microbial species selected by random forest analysis (*n* = 35 species). Results are from covariate-adjusted linear regression models with age, sex, and BMI as independent variables using MaAsLin3. MaAsLin3 displays the two variables with the most significant associations as forest plots (age, Nreads), and the others (sex, BMI) as heatmaps. Results from prevalence data (presence versus not) and those from relative abundance data are displayed. In the forest plots, dots to the right of the zero line are positively associated and those to the left negatively associated with the respective species. Green color refers to prevalence and purple color to abundance with darker shades referring to lower FDR-adjusted p-values. In the heatmap, the colors represent coefficient values and stars FDR-adjusted p-values.

Among the 35 species selected by random forest, prevalence of 14 species associated significantly with age, with 11 being more prevalent in younger participants, and 3 in older ones (Fig. 7). The age-associated microbiome differences reflected distinct ecological patterns. Species more prevalent in younger participants were predominantly facultative anaerobic organisms, including caries-associated taxa such as *Veillonella denticariosi*, and a phylotype each in *Saccharimonadales bacterium* (formerly TM7) and *Streptococcus*. In contrast, species enriched in older participants were strictly anaerobic organisms, including *Alloscardovia omnicolens*, a phylotype in *Fusobacterium*, and *Leptotrichia* (Fig. 7). Beyond the prevalence pattern, we identified 15 species whose relative abundance (when present) significantly associated with age. These abundance associations followed a similar ecological pattern, with facultative anaerobic species like *Granulicatella adiacens* and *Saccharibacteria [F2 G1] bacterium* showing higher abundance in younger participants, while anaerobic species including phylotypes in *Leptotrichia*, *Fusobacterium*, and *Riemerella* increased with advancing age (Fig. 7).

Sex-based differences in the microbiome were less pronounced than age-related variations but still present. Five species showed higher prevalence in men, with none showing higher prevalence in women. Regarding abundance patterns, 8 species were enriched in men (including prevalent *Lancefieldella parvula and* especially in younger men *Oribacterium sinus*) and 9 in women (including prevalent *Haemophilus parainfluenzae* and *Streptococcus infantis*). Only two species (in men) showed consistent associations in both prevalence and abundance analyses (*Alloscardovia omnicolens *and *Lancefieldella rimae*) (Fig. 7). In this study, only a negative association for *Stomatobaculum* *sp.* *HMT097* prevalence and positive associations for *Abiotrophia defectiva* and *Streptococcus sp. HMT423* abundances were observed with BMI (Fig. 7). As expected in microbiota studies, sequencing depth was significantly associated with species prevalence for many taxa. Interestingly, approximately one third of the species also showed abundance associations with sequencing depth, although with inconsistent directions of association (Fig. 7). These technical associations highlight the importance of including sequencing depth as a covariate in microbiome analyses.

### Sensitivity analyses

Sensitivity analyses predicting tobacco use (smoking/snus), oral hygiene practices (toothbrushing frequency, flossing), and self-reported caries susceptibility from metabolome, sugar, or microbiome profiles revealed no significant associations after FDR correction (Supplementary Table 6). However, nicotine metabolite levels were significantly higher in tobacco users in age and sex adjusted glm models. Hence, mean (95% CI) levels in present smokers and/or Swedish moist snus (snuff) users was 2.2 (1.6, 2.7), in those with past use 1.9 (1.6, 2.1) compared to – 0.25 (– 0.32, – 0.17) in never users (*p* = 2.7E-55), respectively, supporting the validity of the self-reported questionnaire reponses.

### Integrated multi-omics modeling identified improved age- and sex-predicting signatures

Integrating all RF-selected features-of-interest from the omics layers did not improve prediction performance for age compared to the LC-MS profile alone but increased prediction of sex slightly (Table 2 versus Table 3 upper part). Restricting the RF model to variables that were significant in the covariate-adjusted linear regressions (Figs. 5, 6 and 7) enhanced prediction of age (Q^2^-values) slightly compared to models using original RF-selected features of interest (Table 3). The finding was supported by a similar increase in Q²-values from 0.67 to 0.74 (*p* < 0.0001) applying OPLS modeling. For sex, a similar improvement in prediction performance was observed after variable restriction with a BER-value reduction from 0.27 in the original model to 0.19, with additional support in BER-value decrease in OPLS-DA to 0.11 (*p* < 0.0001, Table 3). Together this suggests redundance among the variables originally selected, although we cannot exclude the possibility of increased association strength due to data leakage.

The influential features for age prediction based on the final RF model and corresponding OPLS variable selection included 19 LC-MS features/metabolites (including caffeine and trigonelline), 1 sugar (xylonic acid) and 1 bacterium (*Alloprevotella sp*. *HMT914*). For sex prediction, the respective numbers were 5 LC-MS features/metabolites (including creatinine, pantothenol, and lenticin), 2 sugars (ribose and glyceric acid), and 3 bacteria (*Streptococcus sp*. *HMT074*, *Riemerella sp. HMT322* and *Lachnoanaerobaculum umeaense HMT107*) (Supplementary Table 7).

**Table 3:** Performance comparison of integrated multi-omics models for predicting age and sex using random forest and OPLS modeling. Models were trained on participants with complete data across all three omics layers (*n* = 191). Variables were restricted either to those selected from MUVR-RF analysis (1st selection) or to those remaining significant after covariate-adjusted linear regression (2nd selection).

Integrated omics	Number of features			Age		Sex	
	LC-MS	Sugars	Species	Q^2^	*p*-value	BER	*p*-value
1st selection from RF							
RF	85	20	35	0.72	< 0.0001	0.27	< 0.0001
OPLS	85	20	35	0.67	< 0.0001	0.16	< 0.0001
2nd selection from adjusted LM							
RF age/sex	41/19	13/5	29/22^1^	0.74	< 0.0001	0.19	< 0.0001
OPLS age/sex	41/19	13/5	29/22^1^	0.74	< 0.0001	0.11	< 0.0001

LM=linear regression; Q² = proportion of variance predicted in cross-validation; BER = balanced error rate.

^1^Including prevalence and relative abundance.

### Correlation between saliva sugars and bacteria

**Fig. 8:**
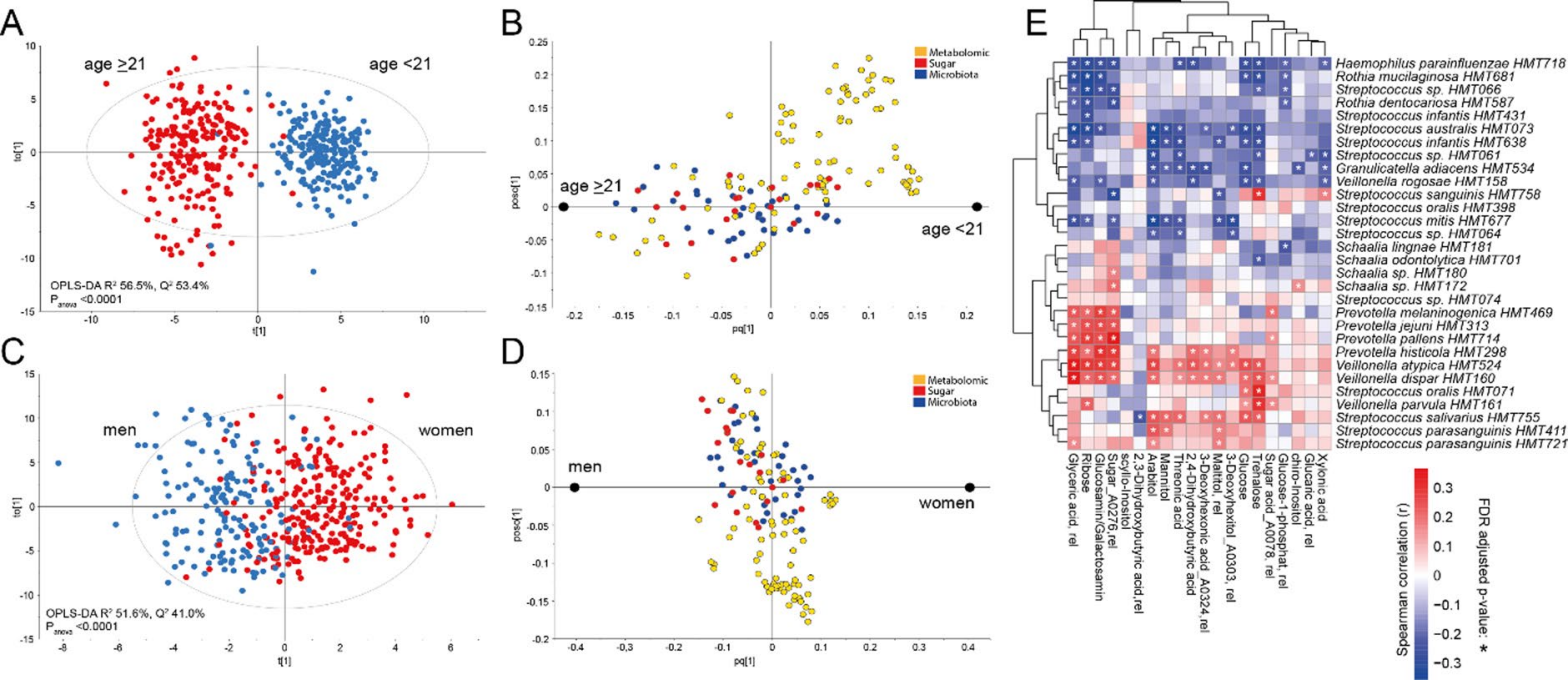
Integration of sugar and microbiome data showing functional relationships. (**A**–**D**) OPLS loading plots demonstrating co-variation patterns between sugar compounds and microbial species in models predicting (**A**, **B**) age and (**C**, **D**) sex. Variables clustering together indicate similar demographic associations. (**E**) Correlation matrix showing partial Spearman correlations between sugar compounds (*n* = 20) and microbial species (*n* = 35), adjusted for age and sex as covariates. Color intensity indicates correlation strength (red = positive, blue = negative). Strong correlations suggest potential metabolic interactions between specific bacterial taxa and sugar metabolism. Stars indicate a p-value of less than 0.05.

A substantial overlap was observed between bacteria and sugars in the OPLS models for both age and sex (Fig. 8A and B), warranting further Spearman correlation analysis between the 20 RF-selected sugars and 35 microbial species. Particularly strong associations were seen for several species in the *Prevotella*,* Rothia*,* Schaalia*,* Streptococcus*, and *Veillonella* genera (Fig. 8C). Notably, several species within the same genus demonstrated similar correlations to the sugar profiles, such as *Prevotella*,* Schaalia*, and several clusters of *Streptococcus* species. However, it was also apparent that several species within the same genus exhibited divergent sugar correlations: e.g. *Veillonella rogosae* had a virtually opposite sugar correlation profile compared to the other *Veilonella* species. Also, the three different *Streptococcus* clusters had distinctly different sugar correlation profiles (Fig. 8C). These opposing trends highlight the functional specialization of oral taxa that genus-level analyses might obscure.

## Discussion

In this study, we present one of the first comprehensive, multi-omics analyses of saliva in a relatively large healthy adult population, combining LC-MS-based metabolomics, targeted and untargeted sugaromics, and full-length 16S rRNA gene microbiome profiling. Our analyses of 200–420 individuals covering a 63-year age span, revealed age as the strongest demographic determinant of saliva molecular profiles across all three omics layers, while sex showed moderate associations and BMI contributed minimally. Multi-omics integration clustered younger participants distinctly apart from older participants, and, with a minor overlap, males from females and improved prediction of the host demographics age and sex slightly compared to single omics. While previous smaller studies^42^ have reported age and sex differences in LC-MS saliva metabolite profiles, the present work is the first to comprehensively integrate saliva metabolome with sugar profiles and microbiome data in a large population, offering novel insights into saliva’s molecular composition and potential as a non-invasive biomarker source.

Our saliva-based multi-omics profiling revealed considerable inter-individual variation in metabolite, sugar, and microbiome features, with distinct clustering across the omics layers by demographic characteristics. While multiple features were significantly associated with age and sex, no consistent associations were observed with BMI. The lack of detectable associations between BMI and saliva metabolome, sugar profile, or microbiome was unexpected and contrasts with previous reports demonstrating BMI-related microbial and metabolic differences^43^. The narrow BMI range and low prevalence of obesity in the present cohort may have yielded a low signal-to-noise ratio and limited the possibility to trace relationships. Nonetheless, several of our findings regarding age and sex align with earlier studies, although direct comparisons are complicated by differences in saliva collection protocols and analytical methods.

Age emerged as the strongest explanatory factor for variance across all molecular layers and it was also the most accurately predicted demographic trait by the RF-selected host- and microbe-derived omics features as well as exogenous compounds, such as food constituents. For instance, positive associations between age and both caffeine-related metabolites and Leukotoxin A (C18H32O3), a well-known secreted isoleukotoxin produced by the anaerobic bacterium *Aggregatibacter actinomycetemcomitans*^44^, are consistent with prior studies linking these features to caffeine intake^45,46^ and periodontitis-associated biofilms^47^, respectively. Notably, the age impact on saliva caffeine metabolites may, beyond intake, be influenced by enhanced plasma leakage via age-associated gingival pocket deepening. Conversely, urocanic acid, a histidine metabolite abundant in the skin and reported to be elevated in the gut of food-sensitized children^48^ and in saliva of patients with Sjögren’s syndrome^49^, was inversely associated with age. We lack information on allergy or hypersensitivity in the study group, but it is noteworthy that the taxa most strongly enriched in younger individuals, including *Saccharimonadales [F2 G1] bacterium HMT989*,* Saccharibacteria (TM7) [G-7] HMT989*), *Caldilinea aerophila*, and *Ignavibacterium album*, also showed the strongest positive correlations with saliva urocanic acid levels (all *p* < 0.001), suggesting a possible interaction and link to immune regulation that warrants further exploration. A broad mapping of metabolites using 18 platforms and various types of tissues, including LC-MS profiling of saliva from 391 participants, was recently presented^50^. While caffeine and trigonelline were detected in both our study and the previous research, the earlier study reported no saliva metabolites with significant demographic associations when applying a stringent statistical threshold (p-value < 10⁻⁸). Among the sex-related differences in our study, elevated levels of pantothenol in females likely reflect cosmetic use^51^, and higher levels of 2,3-dihydroxybutyric acid, creatinine, and isovalerylcarnitine in men confirm previous sex-based differences from blood metabolomics studies^50,52,53^. Beyond the metabolites aligning with previous studies, several novel annotated and unknown metabolites were distinctly associated with age or sex, and several identified metabolites, such as urocanic acid^49,54^, and trehalose^55^, have been linked to disease risk. However, their relevance and mechanistic roles in saliva remain unclear and merit further investigation.

The overall microbiome profile in our cohort was consistent with previous large-scale saliva microbiome studies, including dominance of *Streptococcus*, *Prevotella*, *Veillonella*, *Rothia*, and *Neisseria* and three clusters characterized by species in *Neisseria*,* Prevotella*, and *Streptococcus*^56^. Age-related shifts were observed, with younger participants harboring more saccharolytic, facultative anaerobic, and reportedly caries-associated species, while older individuals showed increased prevalence of anaerobic taxa reportedly linked to periodontal disease in observational and experimental studies^57^. Hence, the observed age-shift might reflect biofilm transformations associated with slowly progressing age-related periodontitis progression though this was not confirmed here due to lack of clinical dental information. Several species within *Streptococcus*, *Veillonella*, and *Prevotella* correlated with sugar levels, with the RF-selected *Prevotella* and *Veillonella* species being positively associated with glyceric acid, glucosamine/galactosamine and ribose. Both *Prevotella* and *Veillonella* species are known to express glycosidases facilitating hydrolysis of glucosides and glucans from complex carbohydrates. Further, *Prevotella* is a key bacterium for fiber degradation in the gut, and the present finding (ribose) opens speculation whether this could be initiated already in the mouth. In contrast, *Streptococcus* species appear more metabolically divergent, as some associate positively with glyceric acid, glucosamine/galactosamine, ribose, arabitol, and trehalose, while others show negative associations. These patterns support the concept that oral microbial ecology and metabolic output are tightly linked, and that demographic factors, especially age, affect both the composition and function of the salivary microbiome. Hence, the observed strong correlations between specific sugars and taxa such as *Streptococcus*,* Prevotella*, and *Veillonella* suggest potential metabolic cross-feeding or niche specialization, warranting further investigation using functional prediction tools, metagenomics, or metatranscriptomics to elucidate the underlying mechanisms. However, such studies need to define the microbiome at least at the species level.

Our study has several strengts and limitations. A major strength of the present study includes the combination of three advanced omics platforms: LC-MS metabolomics, relative and quantitative sugaromics, and full-length 16S microbiome profiling in one of the largest healthy adult saliva omic cohorts to date. A significant challenge is the complexity posed by comparisons across available studies with vast variations in saliva type and collection conditions, demographic characteristics, and analytical methods. Our study utilized stimulated saliva, which differs from unstimulated saliva in both metabolome^58^ and microbiome^59^ profiles, reflecting differences in proportions secreted from different salivary glands and of non-saliva components, but is considered to more comprehensively capture microorganisms at various niches in the mouth than when drooling saliva is used^59^. Specificially, chewing stimulation increases salivary flow and alters ionic composition (e.g., bicarbonate and sodium), while potentially diluting other components due to increased water content. In addition, mechanical shear forces during chewing promote release of microorganisms, epithelial cells, and biofilm material from both tooth surfaces and soft tissues. Age-related changes in salivary gland function, medication use, systemic conditions and diurnal fluctuations may further modulate these effects, which has been extensively described in the literature. Another limitation is that a significant proportion of LC-MS metabolomic features remain unidentified, reflecting current limitations in spectral databases, particularly for salivary metabolites.

Notably, recruitment pathway was closely aligned with age in the present study which is a limitation that makes it difficult to disentangle age effects from recruitment context and therefore suggest observed associations should be interpreted as age- and demography-associated, rather than causal effects of age or recruitment source per se. However, all demographic and behavioral variables that differed between recruitment groups were included simultaneously as covariates in the machine-learning models, ensuring that model estimates were adjusted for these differences and reducing the likelihood that recruitment-related variation drove the observed associations. Moreover, the lack of oral coherent health status data for the entire dataset is a limitation that makes it difficult to fully explain observed associations, such as the age-associated effects on the OMICs-layers which might be explained by oral status and should be further explored in future studies where such data is comprehensively available. Finally, being a cross-sectional observational study, and because regression-based filtering was performed using the full dataset prior to integrative modeling, performance estimates from these models may be optimistically biased, the findings reported here should be interpreted with caution, and further research is necessary to determine the causality and biological relevance of observed associations.

In conclusion, our integrative analysis highlights the substantial impact of age, and to a lesser extent, sex, on the salivary metabolome, sugaromics, and microbiome, with minimal influence of BMI in this healthy cohort. The observed correlations between specific sugars and microbial taxa underscore the value of multi-omics approaches for unraveling oral microbial ecology and its links to host metabolism. Due to its non-invasive collection procedure, saliva has distinct advantages for research involving sensitive populations like children, the elderly, or frail individuals. Our findings provide a critical baseline for future saliva-based biomarker studies and suggest that integration of several omics layers may provide limited improvements in prediction but better possibilities to disentangle molecular mechanisms and determinants of perturbations, thereby providing specific targets for interventions and prevention program for oral and systemic health. However, demographic factors must be carefully considered in study design and data interpretation. Further research incorporating functional prediction, dental status, and longitudinal sampling will be essential to elucidate mechanisms underlying these associations and their implications for oral and systemic health. Future studies should also focus on establishing rigorous standardized collection protocols to ensure validity and reliability.

## Supplementary Information

Below is the link to the electronic supplementary material.

Supplementary Material 1

Supplementary Material 2

## Data Availability

Anonymized data described in this manuscript are uploaded to Figshare, accession number 10.6084/m9.figshare.29966248, and the 16S rRNA sequences are accessible through the BioProject ID PRJNA1332424 (https://www.ncbi.nlm.nih.gov/bioproject/PRJNA1332424) at the NCBI. Additional data can not be shared due to the sensitive personal data of the subjects, which cannot be completely anonymized. These data hence fall under the General Data Protection Regulation (GDPR), which requires restricted access only to authorized personnel with several protection measures. The handling of participants’ personal data linked to their metabolome and microbiome profiles was kept pseudonymized throughout the whole procedure and performed at the national computational infrastructure designed for computations of sensitive personal OMICs data at the server Bianca, provided by NAISS-SENS. Interest in the access and use of additional data or material is welcomed by submission of a written proposal to professor Ingegerd Johansson (ingegerd.johansson@umu.se) or professor Rikard Landberg (rikard.landberg@chalmers.se). R packages used in this study are publicly available.

## Abbreviations

BMI: Body mass index, defined as body weight divided by squared body height (kg/m^2^)
GC-MS: Gas chromatography coupled with mass spectrometry
LC-MS: Liquid chromatography coupled with mass spectrometry
mz: Mass-to-charge ratio
HILIC: Hydrophilic interaction chromatography
RP: Reversed-phase chromatography
