# Supplementary material for: Multi-omics analysis of associations between host demographics and saliva metabolome, sugar profiles, and microbiome profiles
Source: Sci Rep. 2026 Mar 25;16:10494. doi: 10.1038/s41598-026-44287-w (PMC13031898; doi:10.1038/s41598-026-44287-w)
Supplement: Supplementary file 1 — Supplementary Material 1 [file 41598_2026_44287_MOESM1_ESM.docx]

**Online Supplemental Methods**

**Multi-omics analysis of associations between host demographics and saliva metabolome, sugar-, and microbiome profiles**

Stefania Noerman, Anders Esberg, Carina I. Mack, Hany Ahmed, Björn Egert, Elise Nordin, Carl Brunius, Kati Hanhineva, Ingegerd Johansson, and Rikard Landberg

Contents

[Methods 1](#_Toc199487544)

[Saliva metabolome analysis and data processing 1](#_Toc199487545)

[Analysis procedure 1](#_Toc199487546)

[Data processing 2](#_Toc199487547)

[Saliva sugar profiling and data processing 2](#_Toc199487548)

[Combined targeted and untargeted GC×GC-MS analysis 2](#_Toc199487549)

[Data processing 3](#_Toc199487550)

[Saliva microbiota profiling and data processing 4](#_Toc199487551)

[Sample preparation and sequencing 4](#_Toc199487552)

[Sequence data processing 5](#_Toc199487553)

[References 5](#_Toc199487554)

# Methods

## Saliva metabolome analysis and data processing

### Analysis procedure

Metabolomics analysis followed a previously published protocol with minor deviations^1^. Randomized samples (200 µL) were thawed on wet ice prior to vortexing with LC-MS grade ice-cold acetonitrile (400 µL) (Riedel-de Haën™, Honeywell, Seelze, Germany) for 1 min at room temperature. The extracts were centrifuged at 10,600 *g* for 5 minutes at 4°C, and the supernatants filtered through 0.2 µm PTFE filters into a 96-well plate (Nunc™ Deepwell PP, Thermo Scientific, New York, USA). Aliquots (20 µL) were taken from each sample filtrate; mixed to prepare a pooled sample aliquot in one tube and used as a quality control (QC) sample in the data acquisition. An analytical blank devoid of sample material was prepared in the same manner as the samples. The plates were sealed with a 96-well cap mat (Nunc™, Thermo, Scientific, New York, USA) and stored at -20°C until analysis.

The samples were analyzed by a UHPLC-qTOF-MS system (Agilent Technologies, Waldbronn, Karlsruhe, Germany) consisting of an Infinity 1290 II LC system, a Jetstream electrospray ionization (ESI) source, and a 6546 UHD accurate-mass qTOF spectrometer. Each sample (2 µL per injection) was analyzed by two chromatographic techniques, i.e., reversed phase (RP, Zorbax Eclipse XDB-C18 1.8 μm, 2.1 × 100 mm, Agilent Technologies, USA) and hydrophilic interaction (HILIC, Acquity UPLC BEH Amide 1.7 μm, 2.1 × 100 mm, Waters, Ireland) chromatography in both positive and negative ionization mode. The temperature of the sample tray was set at 4°C throughout analysis.

For the RP chromatography, the column oven temperature was set at 50°C and the mobile phase flow rate was set at 400 µL/min for the gradient profile with water (eluent A) and methanol (eluent B, Riedel-de Haën™, Honeywell, Seelze, Germany) both containing 0.1 % (v/v) of formic acid (HiPerSolv Chromanorm™, VWR, Leuven, Belgium). The gradient was 0–10 min: 2% B 🡪 100% B; 10–14.5 min: 100% B; 14.5–14.51 min: 100% B 🡪 2% B; 14.51–16.5 min: 2%. For the HILIC, the column oven temperature was set at 45°C and flow rate at 600 µL/min for the gradient profile with 50 % (v/v) acetonitrile (Riedel-de Haën™, Honeywell, Seelze, Germany) in water (eluent A) and 90 % (v/v) acetonitrile in water (eluent B) both containing 20 mM of ammonium formate (pH 3) (LiChropur™, Supelco, Merck, Darmstadt, Germany).

The MassHunter Workstation Data Acquisition software version 11.0 (Agilent Technologies) (<https://www.agilent.com/en/product/software-informatics/mass-spectrometry-software/data-acquisition/acquisition-for-lc-ms>) was used for data acquisition. Pooled QC samples were injected at the beginning of the sequence and every 12^th^ injection. Instrument parameters were set as follows high resolution (10 GHz) mode, mass range 50–1600 mz, abundance threshold 150 and scan time of 1.67 Hz in the full scan mode. In the data-dependent MS/MS runs, four most intense ions were selected for fragmentation with collision energies of 10, 20 and 40 eV in subsequent runs. Additional parameters for the MS/MS analyses included mass range 50–1600 mz, abundance threshold 200, target 25,000 counts/spectrum, scan time 3.33 Hz, precursor isolation width 1.3 Da, active exclusion after 2 spectra and release after 0.25 min. Source setting for all analyses were the following: drying gas flow 10 L/min and temperature 325 °C, sheath gas flow 11 L/min and temperature 350 °C, nebulizer pressure 45 psi, capillary voltage 3500 V and nozzle voltage 1000 V. Detector was calibrated prior the sample sequence and continuous mass axis calibration was applied by monitoring reference ions mz 121.050873 and mz 922.009798 in the positive mode and mz 112.985587 and mz 1033.98810900 in the negative mode.

### Data processing

Raw instrumental files (.d) were transformed to ABF format using Reifycs Abf Converter (<https://www.reifycs.com/AbfConverter>) for preprocessing of non-targeted metabolomics data. Peak picking and alignment of molecular features were done by MS-DIAL v. 4.92^2^ with the following parameters: MS1 tolerance 0.005 Da, MS2 tolerance 0.015 Da, minimum peak height 5000, minimum peak width 5, retention time tolerances for RP chromatography 0.1 and HILIC chromatography 0.2 min, features detected in at least one group 20 % and gap filling by compulsion was activated. Alignment results were exported as Microsoft Excel sheets to be used in further pre-processing.

Systematic drift corrections and data quality controls were done using the *Notame* v. 0.2.0 R package^1^ in R software v. 4.0.3^3^. Features present in less than 80 % of the sample groups or less than 70 % of QC samples were flagged as low-quality features but kept in the data matrix. Then, log-transformation was applied to all features, and a regularized cubic spline regression line was fitted to each feature against the QC sample to perform correction for signal drift. Leave-one-out cross validation was applied to account for overfitting. Drift correction was applied for all features for each separate mode before the modes were combined. The drift correction was followed by reversal of the log-transformation and features flagged for low-quality if they presented RSD > 0.2 or D-ratio > 0.4. After removing QC samples, random forest imputation was applied for all good-quality features with missing values.

## Saliva sugar profiling and data processing

### Combined targeted and untargeted GC×GC-MS analysis

Frozen saliva samples (both study and QC samples) were thawed on ice, followed by a centrifugation for 20 min at 15000 g and 2 °C (Eppendorf Refrigerated Centrifuge 5403, Hamburg, Germany). 10 µL of an internal standard solution containing 23 isotopically labeled sugar compounds in similar concentrations as expected in the saliva samples (ranging from 0.3 µmol/L to 150 µmol/L, see Supplemental Table S1) were pipetted into a 1.5 mL Eppendorf reaction tube. Before and after addition of 30 µL of saliva, the reaction tubes were weighed, so that the ratio between volume and weight could be used for correction in data analysis. For protein precipitation, 150 µL of ice-cold methanol (Supelco, Sigma-Aldrich, USA) were added and the tube thoroughly mixed. After centrifugation for 15 min at 15000 × g and 2 °C, 120 µL of the supernatant were transferred into a screw-capped glass vial (300 µL fixed insert vial, Thermo Fisher Scientific, Waltham, USA) and evaporated to dryness at 40 °C and 0.1 mbar (p_min_) for 50 min in a rotary vacuum concentrator (RVC 2-25 Cdplus, Martin Christ Gefriertrocknungsanlagen GmbH, Osterode, Germany). Samples were then re-dissolved with 20 µL of methanol and dried again for 20 min at 40 °C and 0.1 mbar (p_min_). Afterwards, a two-step derivatization was performed. For methoximation, 15 µL of a methoxylamin-hydrochloride solution (20 mg/mL dissolved in dry pyridine; Sigma-Aldrich, USA) were added to the dried samples and the reaction was carried out for 1 h at 40 °C and 1000 rpm in a shaker. Afterwards, samples were trimethylsilylated using 50 µL *N*‑methyl-*N*-trimethylsilyl-trifluoroacetamide (Carl Roth GmbH&Co.KG, Karlsruhe, Germany) and the reaction was carried out for 1.5 h at 75 °C and 800 rpm. 10 µL of a retention index marker solution with saturated fatty acid methyl esters (C_7_-C_28_; 250 µmol/L in heptane; Merck KGaA, Darmstadt, Germany) and 10 µL of a retention index marker solution with alkanes (C_8_-C_30_; 250 µmol/L in heptane; Merck KGaA, Darmstadt, Germany) were added to one extra daily QC sample with retention index markers (QR sample). This particular run was employed for identification purposes, facilitating retention index detection and full scan analysis.

For the calibration, seven calibration levels with varying concentrations for each of the 39 selected sugar compounds were prepared. The sugar compounds were categorized into specific concentration ranges, which were anticipated in saliva samples based on preliminary experiments (data not shown). The calibration concentration ranges for different sugar groups were as follows: Threonic acid, arabinose, arabitol, ribitol, psicose, and glucuronic acid: 0.01-1 µmol/L; 2,4-Dihydroxybutyric acid, xylose, xylonic acid, levoglucosan, tagatose, and *chiro*-inositol: 0.01-1 µmol/L; Threitol, erythritol, gluconic acid, and galactaric acid: 0.01-2.5 µmol/L; Arabonic acid, ribonic acid, mannose, glucosamine, and *scyllo*-inositol: 0.05-5 µmol/L; Erythronic acid, sedoheptulose, and trehalose: 0.1-10 µmol/L; 1,5-Anhydroglucitol and *myo*-inositol: 0.75-20 µmol/L; Ribose, xylitol, sorbitol, mannitol, sucrose, and lactose: 0.01-25 µmol/L; Fructose, galactose, *N*-acetyl-glucosamine, and maltitol: 0.5-150 µmol/L; and Fucose, glucose, and maltose: 1-250 µmol/L. In each measurement week, three replicates of each calibration level were randomly measured, interspersed among the study and QC samples. For the calibration levels, 10 µL of the internal standard solution (see above) and 40 µL of the corresponding calibration solution were pipetted into a screw-capped glass vial, followed by the same treatment as the saliva samples, with evaporation and derivatization.

GC×GC-MS analysis of the saliva samples was performed using a Shimadzu GCMS QP2010 Ultra instrument equipped with an AOC-5000 autosampler (Shimadzu Corp, Kyoto, Japan), a PTV injector (OPTIC-4, GL Sciences, Eindhoven, The Netherlands), and a loop-type cryogenic modulator (ZX2, ZOEX Corp., Houston, USA). Table 1 shows information about the consumables, conditions, and specific parameters employed for GC, MS, and modulation. The liner was changed, and the MS was tuned weekly, while the injector septum was replaced after every 100 runs to maintain optimal instrument performance.

Each measurement day, a QR sample with retention index markers, four QC samples, and a total of 17 study and calibration samples (measured in scheduled scan mode 100 Hz) were analyzed. The sequence commenced with a QC sample, followed by the QR sample. Subsequently, three blocks were analyzed, two containing a set of six and one set of five study or calibration samples, interspersed with the remaining QC samples. The QR sample was measured in scan mode, with a data acquisition frequency of 33 Hz and a mass range spanning from mz 50 to 550. On a weekly basis, three replicates of the seven calibration levels were measured.

### Table 1. Method parameters and conditions for sugar profiling using GCxGC-MS.

| **Parameter** | **Setting/value** | |
| --- | --- | --- |
| **Autosampler parameters** | | |
| Injector syringe | 10 µL, with PTFE-tipped plunger (Trajan Scientific, Ringwood Victoria, Australia) | |
| Injection volume | 1.2 µL | |
| Syringe wash solvent 1 | acetone (Merck KGaA, Darmstadt, Germany) | |
| Syringe wash solvent 2 | heptane (Merck KGaA, Darmstadt, Germany) | |
| **GC parameters** | | |
| Carrier gas | Helium 5.0 | |
| GC mode | Constant linear velocity | |
| Purge flow | 6.0 mL/min | |
| Initial column head pressure | 246.0 kPa | |
| Liner type | Deactivated, fritted split liner with quartz wool (CS chromatography, Langerwehe, Germany) | |
| Injector septum | Thermogreen LB-2 (Supelco Merck, Darmstadt, Germany) | |
| Primary column | Rxi-5SilMS, ^1^L = 38,2 m plus 4.7 m of an integrated pre-column,  ^1^d_c_ = 0.18 mm, ^1^d_f_ = 0.36 µm (Restek, Bellefont, USA) | |
| Secondary column | BPX50, ^2^L_total_ = 2.3 m, including a “separation segment” of ^2^L_sep_ = 1.0 m, ^2^d_c_ = 0.15 mm, ^2^d_f_ = 0.15 µm (Trajan Scientific, Ringwood Victoria, Australia) | |
| Column connector | SilTite MicroUnion (Trajan Scientific, Ringwood Victoria, Australia) | |
| GC temperature ramp | 80 °C → 10.00 °C/min → 150 °C → 1.75 °C/min → 220 °C → 8.00 °C/min → 280 °C → 3.00 °C/min → 310 °C → 20.00 °C/min → 320 °C (hold 3.00 min) | |
| Run time | 68 min | |
| Injection mode | Cold split | |
| Split ratio program | 1:5 (hold 1 min) → 1:100 (hold 19 min) → 1:40 (hold until end of run) | |
| PTV temperature ramp | 90 °C → 60 °C/s → 280 °C (hold until end of run) | |
| Interface temperature | 300 °C | |
| **Modulation parameters** | | |
| Modulator type | Cryogenic, air-based, loop-type | |
| Modulation period (P_M_) | 2.1 s | |
| Cold jet temperatur | -90 °C | |
| Hot jet temperatur | 0 min: 200 °C  10 min: → 250 °C  30 min: → 300 °C  55 min: → 360 °C  67 min: → 200 °C | |
| Hot jet pulse duration | 250 ms | |
| **MS parameters** | | |
| Ion source temperature | 200 °C | |
| Ionization mode | EI (70 eV) | |
| MS mode | Scan | |
| Event time | 10 ms | 30 ms |
| Scan speed | 20.000 u/s | |
| Scan range | 8.00 min → 10.55 min: m/z 210-324  10.55 min → 27.19 min: m/z 203-317  27.19 min → 54.09 min: m/z 230-344  54.09 min → 68.00 min: m/z 306-420 | 8.00 min → 68.00 min: m/z 60-550 |
| Data acquisition frequency | 100 s^-1^ | 33 s^-1^ |
| Data acquisition period | 8.00-68.00 min | |

### Data processing

Automated integration with AnalyzerPro XD v1.16.8 (SpectralWorks Ltd., UK) ([spectralworks.com/software-2/analyzerpro-xd](https://spectralworks.com/analyzerproxd-news/)) was used, this included baseline correction, peak detection, noise reduction, and deconvolution. In the next step, files from AnalyzerPro XD were imported and reorganized in a tabular structure, including a conversion of retention times (RT) into ^1^D- and ^2^D-RT (RT in the first dimension/column and in the second dimension/column) based on the modulation time. The result of this step was a first global matrix with a continuous peak list for all runs. Following this, a filtering cascade was applied to achieve a reduction of data, e.g. noise peaks, trace signals and non-sugar compounds were removed. Following the data reduction, a correction of RT shift was performed based on the included isotopically labeled internal standards. Each run was shifted in 4 separate chromatographic regions so that the median of the maximal modulation of all isotopically labeled internal standards was on top of each other. Alignment relied primarily on RT information instead of mass spectral similarity due to the utilization of isotopically labeled internal standards. The process involves three steps: (1) initializing, (2) clustering and splitting, and (3) demodulation. The initializing aims to identify the central modulations corresponding to the same analyte across multiple runs. Combinations of ^1^D- and ^2^D-RT for all peaks in all runs were considered, excluding certain peaks based on specific criteria (peak height, frequency of occurrence, and purity of mass spectra). In the second step, an algorithm clustered neighboring peaks and collects all neighboring peaks that were excluded in the initializing matrix. In the third step, the problem that sometimes clusters may contain multiple closely eluting compounds is addressed. A bimodal split is applied based on variance to separate these compounds. Finally, demodulation is applied and modulations within each cluster are summed up for each run, resulting in a final data matrix for further analysis.

During identification, a specific quantification ion or fragment was selected for each compound (see Supplemental Table S1). The isotopically labeled internal standards were used to correct drift, batch and offset effects. In case of sugar compounds where the corresponding correct internal standard was available, this internal standard was used for correction. In case of sugar compounds where no corresponding isotopically labeled internal standard was available, the best fitting internal standard was selected based on the highest correlation between the signal intensities of the internal standard and the sugar compound within all QC samples (see Supplemental Table S1). The resulting matrix was used for non-targeted statistical data analysis.

For sugars, where a calibration curve was measured, the best fitting regression (linear, linear weighted 1/x, linear weighted 1/x² or polynomial) was selected based on r², residuals and accuracy for QC and additionally measured accuracy samples. The selected regression curve was then used to calculate the concentrations for all saliva samples within the linear range of the calibration curve.

## Saliva microbiota profiling and data processing

### Sample preparation and sequencing

DNA was isolated using GenElute™ Bacterial Genomic DNA Kit (Sigma-Aldrich, St. Louis, MO, USA) from saliva isolated bacteria, a mock community (ZymoBIOMICS Microbial Community DNA Standard (D6305) as positive control), and ultra-pure water (negative control). Briefly, bacteria were harvested by centrifugation for 5 min at 13,000 rpm, lysed in buffer with lysozyme, mutanolysin, and Proteinase K, followed by treatment with RNase. The quality of the extracted DNA was estimated using NanoDrop 1000 Spectrophotometer (Thermo Fisher Scientific, Uppsala, Sweden).

The full-length 16S rRNA gene was amplified using 50 ng input DNA using KAPA 2x HiFi ready mix (New England Biolabs, Ipswich, MA, USA), and the primers 27F 5′-AGA GTT TGA TCM TGG CTC AG-3′ and 1492R 5′-CGG TTA CCT TGT TAC GAC TT-3′. The amplification was performed on a MiniAmp™ Thermal Cycler (Thermo Fisher Scientific) using the program; 1 min denaturation at 98°C, 35 cycles (95°C 20 s, 55°C 15 s, 72°C 1.5 min), and a final extension step of 1.5 min at 72 °C. A single fragment of the expected size was confirmed by separating amplicons on a 0.8 % agarose gel. Sample/amplicon was purified using the AMPure XP Beads (Beckman Coulter, Brea, CA) and quantified using the Qubit dsDNA HS Assay Kit and Qubit 4.0 Fluorometer (Invitrogen, Thermo Fisher Scientific, Oregon, USA).

Library preparations were performed by barcoding full-length 16S rRNA products using Native Barcoding Kit 96 V14 (SQK-NBD114.96) kit (Nanopore) (<https://store.nanoporetech.com/native-barcoding-kit-96-v14.html>) according to manufacturer description. Briefly, 200 fmol PCR products were end-repaired using NEBNext® Ultra™ II End Repair/dA-Tailing Module (NEB, Cat #: E7546L), ligated with unique barcodes for each sample using NEB Blunt/TA Ligase Master Mix (NEB, Cat # M0367). Samples were pooled in batches of 80 and finally fused to the Native Adapter using T4 DNA Ligase (NEB E6056). Barcoded pooled libraries were quantified using a Qubit fluorometer (Life Technologies, Carlsbad, CA).

Sequencing of barcoded pools was performed by loading 100 ng into a primed R10.4.1 flow cell (Oxford Nanopore Technologies—ONT, Oxford, UK) and sequenced using a GridION nanopore sequencer (Oxford Nanopore Technologies—ONT, Oxford, UK) for 72 hrs. The settings “high-accuracy” and reads >1000 bp to be collected and base called by the MinKNOW (MinION software, version 1.6, Oxford Nanopore Technologies—ONT, Oxford, UK) were applied. Raw fast5 files were base-called using Guppy version 6.2.7 to generate fastQ files and demultiplexed and adapters trimmed using Porechop (version 0.2.4). Passed multiplexed fastQ files were processed using the EMU pipeline^4^ with species relative abundance above 0.0001 [--min-abundance 0.0001].

### Sequence data processing

For matching the filtered and quality-checked 16S rRNA sequences, the Emu pipeline (<https://github.com/treangenlab/emu?tab=readme-ov-file>)^4^,^5^ was employed in combination with the curated extended Human Oral Microbiome Database^5,7^ (rRNA gene RefSeq Version 15.23), which contains 1,015 16S rRNA gene sequences covering 774 oral bacterial species (Table 2023-11-03_1698999909). Sequencing depth, alpha- and beta-diversity were assessed using R and RStudio (version 2023.12.1) with the package MicrobiotaProcess^9^ and its integrated dependencies. Functions including mp_rrarefy, mp_cal_rarecurve, mp_plot_rarecurve, mp_cal_alpha, mp_plot_alpha, mp_cal_abundance, and mp_plot_abundance were used to rarefy all samples to equal sequencing depth, and to calculate, evaluate, and visualize taxonomic composition and alpha-diversity metrics such as species richness. Compositional comparisons between sequencing platforms or DNA extraction kits were performed using mp_decostand, mp_cal_dist, mp_plot_dist, mp_cal_pcoa, and mp_adonis functions, applying the Bray-Curtis distance matrix. Feature differences between subgroups were extracted and visualized with mp_plot_ord and mp_diff_analysis.

## References

1. Klåvus A, Kokla M, Noerman S, Koistinen VM, Tuomainen M, Zarei I, et al. "notame": Workflow for Non-Targeted LC-MS Metabolic Profiling. Metabolites 2020;10:135.
2. [Tsugawa](https://pubmed.ncbi.nlm.nih.gov/?sort=date&term=Tsugawa+H&cauthor_id=32541957) H, [Ikeda](https://pubmed.ncbi.nlm.nih.gov/?sort=date&term=Ikeda+K&cauthor_id=32541957) K, [Takahashi](https://pubmed.ncbi.nlm.nih.gov/?sort=date&term=Takahashi+M&cauthor_id=32541957) M, [Satoh](https://pubmed.ncbi.nlm.nih.gov/?sort=date&term=Satoh+A&cauthor_id=32541957) A, [Mori](https://pubmed.ncbi.nlm.nih.gov/?sort=date&term=Mori+Y&cauthor_id=32541957) Y et al.. A lipidome atlas in MS-DIAL 4. Nat Biotechnol. 2020;38:1159-1163.
3. Core Team. R: A Language and Environment for Statistical Computing [Internet]. Vienna: R Foundation for Statistical Computing; 2019. Available from: <https://www.r-project.org>
4. Curry, K.D. et al. Emu: species-level microbial community profiling of full-length 16S rRNA Oxford Nanopore sequencing data. *Nat Methods*. **19**, 845-853 (2022).
5. Curry, K.D. et al. Microbial Community Profiling Protocol with Full-length 16S rRNA Sequences and Emu. *Curr Protoc*. **4**, e978 (2024).
6. Chen, T. et al. The Human Oral Microbiome Database: a web accessible resource for investigating oral microbe taxonomic and genomic information. *Database (Oxford)*. **baq013** (2010).
7. Escapa, I.F. et al. New Insights into Human Nostril Microbiome from the Expanded Human Oral Microbiome Database (eHOMD): a Resource for the Microbiome of the Human Aerodigestive Tract. *mSystems*. **3**, e00187-18 (2018).
8. Xu, S. et al. *MicrobiotaProcess* A comprehensive R package for deep mining microbiome. *Innovation (Camb)*. **4**, 100388 (2023).
9. Curry, K.D. et al. Emu: species-level microbial community profiling of full-length 16S rRNA Oxford Nanopore sequencing data. *Nat Methods*. **19**, 845-853 (2022).
